# The Prevalence of Familial Multiple Sclerosis in Saskatoon, Saskatchewan

**DOI:** 10.1155/2014/545080

**Published:** 2014-02-03

**Authors:** Walter J. Hader, Irene M. Yee

**Affiliations:** ^1^Department of Physical Medicine and Rehabilitation Saskatoon, University of Saskatchewan, Saskatchewan, Canada; ^2^Saskatoon City Hospital, 701 Queen Sreet, Suite 7717, Saskatoon, Saskatchewan, Canada S7K 0M7; ^3^Department of Medical Genetics, Faculty of Medicine, University of British Columbia, British Columbia, 2211 Westbrook Mall, Vancouver, British Columbia, Canada V6T 2B5

## Abstract

*Background*. A population-based prevalent cohort of 150 clinical definite multiple sclerosis (MS) cases (102 women; 48 men) ascertained on January 1, 1977, Saskatoon, Saskatchewan, was found to have a familial rate of MS as 17.3%. *Objectives*. To determine the occurrence of familial MS cases and the frequency of MS among the biological relatives of the study cohort. *Methods*. The search for new familial cases MS affected relatives continued for 35 years until 2012. The natural history of the disease of sporadic cases is compared with that of the familial cases. SPSS V19 and Kaplan-Meier survival analysis were used for data analysis. *Results*. Of the 150 unrelated MS patients, 49 cases (32.7%) (36 women and 13 men) were reported of having at least one family member with MS. There were a total of 86 affected relatives, 26 (30.2%) first-degree relatives, 15 (17.4%) second-degree relatives, 20 (23.3%) third-degree relatives, and 25 (29.1%) distant relatives. The average age of MS onset for men with sporadic MS was 33.9 (SD = 10) years and 27.6 (SD = 8.4) years for familial cases and 29.3 (SD = 8.3) years and 26.8 (SD = 8.5) years for women. *Conclusion*. This 35-year longitudinal natural history study reveals a high frequency of cases with family members developing MS and supports a genetic influence in the etiology of MS.

## 1. Introduction

In the contemporary history of multiple sclerosis (MS), the familial recurrence rate is estimated at about 15% and is variable in geographic locations or absent in some ethnic groups [1, 2]. In the early history of MS, there was mention of some familial cases [3]. This was also seen by Charcot but not mentioned in the 1868 description [4]. The earliest collection and report of familial cases were by Curtius in 1933 [5]. Pratt et al. in 1951 described the first incidence rate of 6.5% in a collection of 310 cases and proposed the concept of polygenetic inheritance [6]. Millar and Allison reported the same incidence in 1954 [7], Sutherland 11% in 1956 [8], and Guodmundsson 14.9% in 1971 [9]. In 1972, Jersild et al. [10] was the first to report an association between the disease and certain alleles of the HLA complex and evidence supporting a genetic factor in MS. Over the next 30 years in larger registries and databases, higher rates are reported by Sweeney et al. in 1986 (20%) [11], Robertson et al. in 1996 (19.1%) [12], and Carton et al. 1997 (17.2%) [13]. The genome-wide association Studies have recently confirmed about 59 genetic variants associated with MS and considered that MS is an immunological disorder [14].

The role of environmental factors and infectious and noninfectious agents that may influence the etiology of MS has not been determined [15, 16]. Pedigree analysis does not fit any particular Mendelian pattern.

The primary objective of this longitudinal natural history study was to determine the frequency of MS cases with occurrence of MS among biological relatives and to compare the clinical characteristics of the sporadic and familial cases. A further aim was to analyze factors that may have effects on the survival of MS.

## 2. Methods

The original cohort of 150 clinical definite MS patients ascertained in a population-based prevalence study in Saskatoon, Saskatchewan, on January 1, 1977, was the basis for this study [17]. These patients were interviewed for their family histories and followed to determine any new family members diagnosed with MS. An MS registry was established in 1969, beginning with a retrospective search of all medical records in the three local hospitals (St. Paul's Hospital, Saskatoon City Hospital, and Royal University Hospital).

Case information was obtained from nursing homes, the Home Care Program, the MS Society of Canada, Saskatoon Chapter, MS Rehabilitation clinic database, family members, physicians, and neurologists. Surveillance for prevalent cases was continued until 1980 to allow for further case ascertainment with onset that occurred before prevalence date. The prevalence of MS in the City of Saskatoon on 1 January 1977 was 110/100,000 for clinical definite cases that are the subjects for this study.

The diagnostic classification, modified from Allison and Millar [18], included probable (clinical definite), possible, and suspect MS, and the diagnostic criteria adapted from those of Schumacher et al. [19] for the probable category. All living patients who had resided in Saskatoon for at least one year were included in the prevalent group. All cases had a confirmed diagnosis by one or more neurologists except one by an internist.

The development of the registry, the search of medical records, and methods of ascertainment were detailed in previous reports [20, 21]. The number of unrelated cases with MS affected relatives was recorded on prevalence day. The familial cases were summarized after the first and second decade. The numbers of familial cases and affected relatives were updated annually, tabulated each five years for a total of 35 years, and concluded on 1 January 2012.

Information including demographic data on gender, date of birth, date of onset, date of diagnosis, onset symptoms, familial history, Kurtzke Disability Status Scale (DSS) [22], course of disease (primary progressive, relapsing-remitting, and secondary progressive, PPMS, RRMS, and SPMS), duration of disease after onset, and date of death was collected and entered into a computer database. Follow-up information was collected by telephone interviews in which patients were asked about new occurrences of MS in their family members. Consent was obtained through the cases to interview new family members and obtained confirmation of diagnosis.

All MS patients living in the local area were accessible except for six women who had moved away and were unable to be located. This study had approval of the Regional Research Ethics Board.

## 3. Statistical Analysis 

The descriptive analysis was performed using SPSS Version 19. The frequency of biological relatives affected with MS was computed. The survival of familial and sporadic MS patients from onset was estimated by the Kaplan-Meier product-limit estimation and the equality of survival distribution was tested by the log-rank (Mantel-Cox) test [23]. The joint relationship of covariates to the mortality of MS was analyzed using the Cox proportional hazards analysis. The analyses yielded estimates of risk factor effects adjusted simultaneously for the effects of all other risk factors in the model. In the present study, six covariates were considered in the Cox proportional analyses: (i) gender, (ii) age of MS onset, (iii) onset symptoms, (iv) MS course, (v) birth cohort, and (vi) family history of MS (more than one affected in a family, yes or no). The survival time from the onset of MS was used as the time variable and the time for the 6 missing cases was from onset of MS to when they were last seen. Appropriate interaction terms were also included in the analysis for evaluation.

## 4. Results 

Of the 150 unrelated clinical definite patients, we found 49 cases (32.7%) with 86 biological relatives with MS after 35 years of follow-up. 13/48 men and 36/102 women had at least one family member with the diagnosis of MS. The average age of onset for sporadic men was 33.9 (SD = 10) years and 27.6 (SD = 8.4) years for familial cases and 29.3 (SD = 8.3) years and 26.8 (SD = 8.5) years for women (*P* = 0.017). The mean duration of disease was 15.5 (SD = 9.5) years for the men and 15.9 (SD = 10.3) years for the women on prevalence date.

Table 1 shows the number of familial cases and affected relatives at baseline and the accruing numbers of familial cases and affected relatives at 10, 20, 25, 30, and 35 years of follow-up. At baseline, 26 (17.3%) of the cohort had 31 affected relatives with MS. After twenty-five years, the number of familial cases increased to 49 and the number of affected relatives increased to 69 cases. From 25 to 35 years of follow-up the number of familial cases (*N* = 49) did not increase, but the number of affected relatives had increased from 69 to 86 cases. Most relatives of the surviving cohort may not be expected to develop MS, because they are beyond the range of onset, averaging over 62 years of age. Only relatives in the younger generations may become affected with MS in the future.

A total of 31 patients have 1 relative, and 18 patients have 2 or more relatives with MS. There were two affected MS female patients with unaffected identical twin sisters and one affected male with an unaffected nonidentical twin brother. There was one conjugal pair with MS. There were two multi-generational families with seven and eight affected family members that had accumulated up to the last decade of the study, 3 had been ascertained in the last 5 years, and these were paternal 2nd cousins.

The two family pedigrees on analysis with the 7 and 8 affected family members over 5 generations do not follow any Mendelian pattern.

The frequencies of affected male and female family members are shown in Table 2. There were a total of 86 affected relatives, 26 (30.2%) first-degree relatives (parents, full siblings, and children), 15 (17.4%) second-degree relatives (aunt, uncle, niece, nephew, and great grandparent), 20 (23.3%) third-degree relatives (first cousins), and 25 (29.1%) distant relatives (second and third cousins). The sex ratio of the 71 female relatives to 15 male relatives was 4.7. There were 6 half-brothers and 8 half-sisters and 8 adopted nonbiological males and 5 adopted nonbiological females, and they were not affected by MS. The recurrence risks are shown for first-degree relatives but not for remaining groups because of the uncertainty of the exact number of 2nd and 3rd degree relatives in each of the family groups.

The results of the Cox analysis suggested that the family history of MS, a binary variable, has a substantial modification effect on the survival of cases as the interaction between MS course and family history as well as age of MS onset and family history was statistically significant. The number of cases and the hazard ratios are shown in Table 3. In the absence of other factors, the presence of family history appeared to prolong the survival of female cases. In the multivariate Cox analysis, for example, cases with SP MS and a family history of MS, the mortality hazard was equal to 6.6 times the hazard of cases with RR MS and a family history of MS.

After 35 years of follow-up, 29 (19.3%) MS patients (9 men and 20 women) have survived, 115 deceased, and 6 cases were missing. There were 16 cases with DSS ≤6 (3 male and 6 female familial cases; 3 male and 4 female sporadic cases) and 13 cases DSS >6 (6 female familial cases; 3 male and 4 female sporadic cases).

There were 15/29 (51.7%) familial cases that were surviving. The age of onset of the 3 surviving men was 26.0 (SD = 6.4 years) and 21.6 (SD = 4.0 years) for the 12 women. The average duration of disease for the living males was 41.3 (SD = 5.5) years and 41.8 (SD = 7.9) years for the females.

The average age of onset of the 6 living sporadic males was 30.2 (SD = 3.0) years and 25.0 (SD = 4.3) years for the 8 females. The average duration of disease for the living males was 43.5 (SD = 3.0) years and 39.5 (SD = 2.9) years for the living females.

The survivorship in years after onset, by comparing gender and familial and sporadic cases of multiple sclerosis, is shown in Table 4. Means and medians and the upper and lower figures represent 95% confidence intervals.

**Figure 1:**
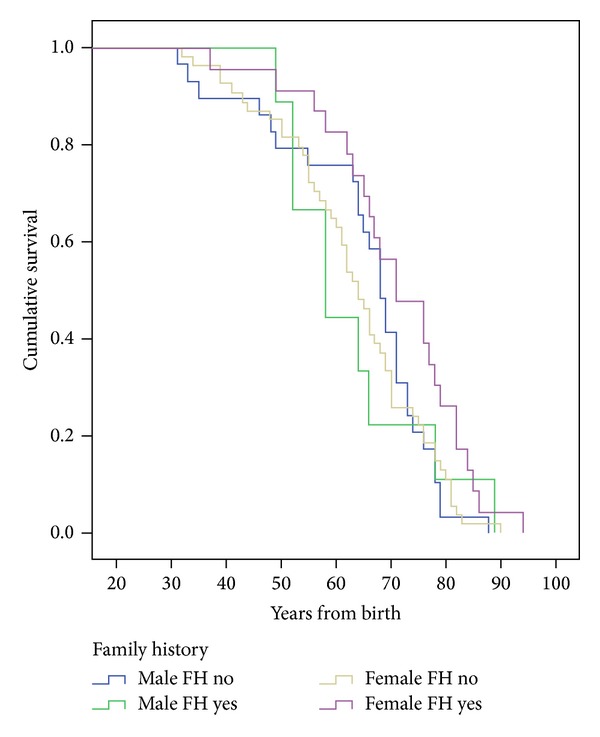
Survivorship in familial multiple sclerosis. Percentage survival by years after onset by gender and comparing different levels of familial and sporadic groups. Log-rank (Mantel-Cox) test, *P* = .000.

The median survival after onset for sporadic men was 33 (95% CI: 26.1–39.1) years and 35 (95% CI: 17.4–52.6) years for familial cases and 36 (95% CI: 32.7–39.3) years and 51 (95% CI: 45.4–56.8) years for the women (see Figure 1). The log-rank test (Mantel-Cox) for equality of survival curves shows a highly significant difference (*P* = .000).

The mean duration of life for sporadic and familial men was 67.3 and 68.0 years and 64.7 and 73.9 years for women. The median survival from birth for the familial and sporadic cases was 76 (95% CI: 67.9–84.2) and 66 (95% CI: 61.9–70.1) years for the women and 69 (95% CI: 65.8–72.2) and 66 (95% CI: 50.4–77.2) years for the men (*P* = 0.031).

The Canadian life expectancy for men is 76.7 years and 82.3 years for women with a significant difference of 7.7 years less for the men and 6.3 years less for women [20] in this study.

## 5. Discussion

The purpose of this study was to investigate the frequency of persons affected by MS with affected family members as well and to compare the natural history of the disease among sporadic and familial cases.

This longitudinal family history study with 35 years of follow-up has identified a high familial rate (32.7%) in a population-based prevalent cohort of clinical definite patients that provides evidence for genetic susceptibility to MS. The cohort numbers are small but it is a representative group of sporadic and familial cases. The main strength is that this is a population-based prevalent cohort and the longitudinal follow-up by the same investigator since the beginning of the study. In the Saskatoon report, on prevalence day, there were 26 of 150 patients with 31 affected relatives (17.3%) that have increased after 35 years of follow-up to 49 of 150 (32.7%) patients that have 86 relatives diagnosed with MS. The long-term surveillance has determined a high frequency of familial MS. In the last 10 years of the study, 17 new affected relatives were ascertained, primarily distant relatives that would not have been found in any shorter term studies.

After 30 years of follow-up, 105 (70%) of patients had deceased and no further information was available about additional affected relatives. At the 35th year of follow-up, 115 MS patients had deceased and 29/35 of living patients on interview provided no new relatives with MS. The high frequency of cases may represent the maximum familial rate of MS but yet further relatives in the younger generations may be affected in future.

The Middlesex County 25-year population-based study identified a high familial rate of 22.5%, an increase of 17% in a previous 15 year study [24]. Ebers et al. [25] have stated that the results in the follow-up study have established a natural history of approximately 20% of patients with familial disease, and that patients with familial disease have a clinical course that differs a little from sporadic cases and are not distinct from each other. This study reveals that after longer follow-up there is an increasing frequency of new persons with MS among family members and that there are differences in the clinical course and survival between the genders of the sporadic and familial cases. The number of new index cases (*N* = 49) seems to have peaked after 25 years, but the number of new affected relatives in these index cases increased from 69 to 86 cases in the last decade and three 2nd degree cousins were reported in the last five years, thus emphasizing the need for long-term longitudinal studies to determine the frequency of familial MS.

The recurrence risks of relatives are variable in reports [12, 13, 26]. In this study, there were no identical twins with MS, and the one affected conjugal pair had no children. There were no affected cases in the half-siblings and nonbiological adopted children related probably due to the low numbers in the families or not yet affected. The mother/daughter risk is similar to the report of Sadovnick [27] but mother/son and father/son risks are found in this study. The age-adjusted recurrence risks range from 2 to 5% in the first degree relatives. A maternal parent-of-origin effect in MS has been proposed by Herrera et al. [28].

The variability and effect of different factors on the survival outcomes between males and females are not clearly defined. Weinshenker [29] states that the clinical factors associated with more favorable outcomes including younger age of onset, female gender, relapsing-remitting course of disease, late onset of disease, and progressive course of disease are detrimental to survival. In the Saskatoon study [21] five covariates were considered in the Cox proportional analysis and showed strong evidence for a relation between age of onset and the course of the disease (RR). In this study, the familial factor was added to determine any effect on survival.

Without the presence of other factors, there appears to be a distinct difference in survival from onset between the genders and the familial and sporadic cases. The familial characteristic and an earlier age of onset in women seem to have an influence on prolonging survival of MS. When using the Cox analysis to consider the joint effect of other covariates, the results indicate that the presence of family history of MS has a detrimental effect on the survival of cases with more progressive course of MS and later onset of MS.

In a previous report [20], the life table analysis estimated a median survival of 68.95 (95% CI: 65.4–72.6) years for men and 69.5 (95% CI: 65.5–72.6) years for women, a significant difference of 7.7 years less for men and 12.8 years for women compared to Canadian normal population. This report with the addition of the familial factor shows the men median survival of 69 years and a significant increase to 76 years for women. The median survival from birth for sporadic cases of both genders was 66 years, a significant difference compared to the familial cases. The familial factor appears to have an influence on survival from onset and longevity.

The average duration of disease on prevalence date was 15 years and this natural history study covers a further period of 35 years and therefore provides more long-term information on the familial occurrence, the disability outcomes, and the new observation that there appears to be also a familial influence on survival outcomes and longevity of the disease.

The first evidence of a genetic risk factor for MS in the HLA complex was first identified by Jersild et al. [10] Genome-wide association studies have since confirmed 59 loci that play a key role in disease susceptibility and clearly confirmed that the HLA DRB1 ∗ 15.01 risk alleles have the strongest association with multiple sclerosis [14]. Population-based studies have shown that the recurrence rate of MS ranges from 20 to 30% in monozygotic twins and 5% in dizygotic twins [30]. Further lines of evidence of genetic factors are the greater risk of biological first degree relatives over the general population, decreased risk in half siblings, increased risk in siblings in conjugal pairs [31], multigenerational families of MS [32], and the founder effect in genetic isolates [33, 34].

## 6. Conclusions

This long-term study provides unique information in a population-based prevalence cohort on the high frequency of familial cases in MS. The familial cases compared to the sporadic cases show a significant difference in survival from onset and duration of life between men and women. This natural history of 150 unrelated MS patients reveals a high aggregation of familial cases and supports a genetic influence in the etiology of MS. However, this observation was only possible over an extended period of 35 years.

Newly developed technologies in genetic analysis may provide additional evidence for genetic susceptibility in MS more efficiently, but it still requires long-term follow up to truly know the MS status of relatives, especially in the younger generation.

## Figures and Tables

**Table 1:** Familial multiple sclerosis. Frequencies and percentage of total familial cases, the number of affected relatives in each family, and the total number of relatives, in the years of follow-up.

	Number of affected relatives in family								
	1	2	3	4	6	7	8	Total relatives	Total cases/150
Number of families	22	3	1	—		—	—	31	26 (17.3%)
Years of follow-up									
10 years	22	5	1	—		—	—	35	28 (18.7%)
20 years	28	8	5	1	—	—	—	63	42 (28.0%)
25 years	31	12	5	1		—	—	69	49 (32.7%)
30 years	31	10	4	2	2			83	49 (32.7%)
35 years	31	10	4	2	—	1	1	86	49 (32.7%)

**Table 2:** Frequency and relationships of male and female close (1st, 2nd, and 3rd degree relatives) and distant (2nd and 3rd cousins ) affected relatives and recurrent risks of 1st degree relatives.

	Male	Female	Total	Recurrence	Risks	Percentage
Brother		7	7	Brothers	7/269	2.6
Sister	1	5	6	Sisters	6/276	2.2
Son	*3 *	*3 *	*6 *	Mother/son	*3/138 *	*2.2 *
Daughter		7	7	Mother/daughter	7/127	5.5
Paternal grand father		1	1	Father/daughter	—	—
Paternal uncle		1	1	Father/son	3/138	2.2
Maternal aunt	1	1	2	Siblings	13/265	4.9
Paternal aunt		3	3			
Maternal niece		2	2			
Paternal niece	1	2	3			
Maternal nephew		2	2			
Paternal nephew		1	1			
paternal 1st cousin	3	5	8			
2nd cousin		15	15			
3rd cousin		1	1			
maternal 1st cousin	5	7	12			
2nd cousin	1	6	7			
3rd cousin		2	2			

Total	15	71	86			

**Table 3:** Cox proportional analysis: hazard ratios for covariates and interaction terms.

Covariates	Hazard ratio	*P* value
MS course		
Relapsing-remitting (RR)	1.0	
Secondary progressive (SP)	1.5 (95% CI: 0.9–2.4)	0.14
Primary progressive (PP)	3.4 (95% CI: 1.7–6.9)	0.00074
Family history of MS (FHMS)		
No	1.0	
Yes	0.03 (95% CI: 0.005–0.2)	0.00012
Age of MS onset		
<20	1.0	
20–29	0.5 (95% CI: 0.2–1.2)	0.11
30–39	0.7 (95% CI: 0.3–1.9)	0.51
≥40	0.9 (95% CI: 0.3–2.6)	0.90
MS course × FHMS (yes)		
RR × FHMS	1.0	
SP × FHMS	6.6 (95% CI: 1.8–23.7)	0.0037
PP × FHMS	2.5 (95% CI: 0.6–9.5)	0.19
Age of MS onset × FHMS (yes)		
Onset <20 × FHMS	1.0	
Onset 20–29 × FHMS	5.4 (95% CI: 1.0–29.0)	0.052
Onset 30–39 × FHMS	3.2 (95% CI: 0.5–18.9)	0.20
Onset ≥40 × FHMS	23.2 (95% CI: 3.6–149.5)	0.00093

**Table 4:** Survival in years after onset, by gender and familial and sporadic cases of multiple sclerosis. Means and medians estimates and upper and lower figures are 95% confidence intervals.

Family (FH)	History	Means				Medians			
		Estimate	Std error	95% confidence intervals		Estimate	Std error	95% confidence intervals	
				Lower	Upper			Lower	Upper
Male	FH no	33.168	2.011	29.226	37.109	33.000	3.537	26.068	39.932
	FH yes	40.385	4.131	32.289	48.481	35.000	8.987	17.385	52.615

Female	FH no	34.234	1.592	31.114	37.354	36.000	1.692	32.683	39.317
	FH yes	46.336	2.291	41.847	50.826	51.000	2.846	45.422	56.578

Overall		37.565	1.173	35.266	39.863	37.000	1.662	33.742	40.258

Log-rank (Mantel-Cox) test, *P* = .000.
